# Pathological Complete Response after Pembrolizumab Treatment for Unresectable Perihilar Cholangiocarcinoma with High Microsatellite Instability: A Case Report

**DOI:** 10.70352/scrj.cr.25-0025

**Published:** 2025-04-25

**Authors:** Yoshikuni Inokawa, Hironori Mizuno, Mihoko Yamada, Shoji Kawakatsu, Nobuyuki Watanabe, Shunsuke Onoe, Takashi Mizuno, Kohei Okayama, Fumihiro Okumura, Masaki Kajikawa, Tomoki Ebata

**Affiliations:** 1 Department of Surgery, Gifu Prefectural Tajimi Hospital, Tajimi, Gifu, Japan; 2 Department of Surgery, Nagoya University Graduate School of Medicine, Nagoya, Aichi, Japan; 3 Department of Gastroenterology, Gifu Prefectural Tajimi Hospital, Tajimi, Gifu, Japan

**Keywords:** MSI-high, TMB-high, pathological complete response, perihilar cholangiocarcinoma, biliary tract cancer, pembrolizumab, conversion surgery

## Abstract

**INTRODUCTION:**

Pembrolizumab has been introduced to solid cancers with microsatellite instability (MSI)-high cases; however, its clinical experience for cholangiocarcinoma remains very limited. Here, we present a case who successfully underwent conversion surgery following pembrolizumab treatment for MSI-high perihilar cholangiocarcinoma, which pathologically exhibited complete response.

**CASE PRESENTATION:**

A 69-year-old male with Bismuth IV perihilar cholangiocarcinoma with bulky lymphadenopathy was referred, who initially required left hepatic trisectionectomy, caudate lobectomy, bile duct resection, and portal vein resection and reconstruction (H123458-B-PV). During the waiting period after preoperative portal vein embolization, the right hepatic artery was involved by rapid tumor progression, needing a modification of the initially scheduled surgical procedure to additional hepatic artery resection and reconstruction (H123458-B-PV-HA). We revised the surgical decision of resectable to locally unresectable disease. He received systemic chemotherapy with gemcitabine and cisplatin as first-line, showing the best effect of stable disease followed by slight tumor progression and re-elevation of tumor marker after 5 courses of treatment. Cancer multi-gene panel analysis using percutaneous biopsy specimen showed the nature of MSI-high. Therefore, he received pembrolizumab treatment as second-line therapy, leading to a drastic downsize >30% in tumor diameter and normalization of the tumor marker as well after only 2 cycles of administration. After confirmation of keeping tumor shrinkage during 22 courses of pembrolizumab treatment without any severe adverse events, we decided to perform conversion surgery and performed left trisectionectomy, caudate lobectomy, and bile duct resection with portal vein resection (H123458-B-PV). Although the right hepatic artery was extensively fibrotic, there was no evidence of malignancy by frozen section histologic diagnosis. The pathological findings showed pathological complete response with no residual tumor cells. The patient is under periodical checkup without adjuvant chemotherapy, and no tumor recurrence was observed at 4 months postoperatively.

**CONCLUSIONS:**

We experienced clinical partial response but pathological complete response after second-line pembrolizumab treatment for unresectable locally advanced perihilar cholangiocarcinoma with a biologic nature of MSI-high. Conversion surgery may be considered as a promising option for such effective case, whereas there is a possibility to avoid resection in the MSI-high setting.

## Abbreviations

^18^F-FDG PET-CT
: fluorin-18 fluorodeoxyglucose positron emission tomography–computed tomography
BTC
: biliary tract cancer
CA19-9
: carbohydrate antigen 19-9
cCR
: clinical complete response
CT
: computed tomography
FGFR2
: fibroblast growth factor receptor 2
GCD
: gemcitabine/cisplatin/durvalumab
GCP
: gemcitabine/cisplatin/pembrolizumab
MSI
: microsatellite instability
pCR
: pathological complete response
PD-1
: programmed cell death 1
PD-L1
: programmed cell death ligand 1
PTPE
: percutaneous transhepatic portal vein embolization
TMB
: tumor mutational burden

## INTRODUCTION

The prognosis of biliary tract cancer (BTC) is still poor because of frequent locally advanced or metastatic disease at initial diagnosis, which often precludes curatively intended resection.^1)^ Recently, several multi-drug regimens for BTC have been established in the unresectable or recurrent setting,^2–7)^ however, the treatment option remains limited. Pembrolizumab, a humanized anti–programmed cell death 1 (PD-1) inhibitor, is currently used for advanced solid tumors limited with microsatellite instability (MSI)-high and tumor mutational burden (TMB)-high.^8–10)^ The prevalence is very rare with a reported incidence ranging from 2% to 9% in BTC,^11,12)^ therefore, the clinical course with pembrolizumab for BTC remains poorly acknowledged.

We experienced a case with unresectable locally advanced perihilar cholangiocarcinoma with MSI-high and TMB-high who received long-term pembrolizumab treatment. The focus of interest was that successful resection was completed and the tumor showed pathological complete response (pCR).

## CASE PRESENTATION

**Fig. 1:**
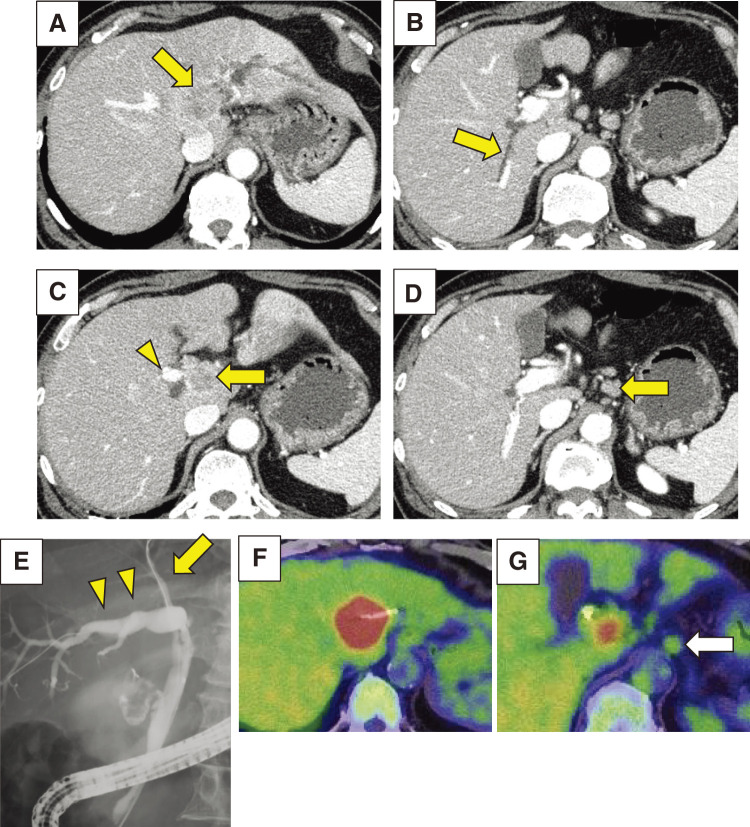
Findings before chemotherapy. (**A**) Dynamic enhanced CT at initial assessment showed a perihilar tumor (arrow), 27 mm in diameter, with an upstream dilatation of the intrahepatic bile ducts. (**B**) Posterior bile duct was dilatated (arrow) in the same enhanced CT. (**C**) Anterior branch of portal vein (arrowhead) was adjacent to the tumor (arrow). (**D**) Bulky lymphadenopathy (arrow) was detected by the same enhanced CT. (**E**) ERCP showed obstruction of left hepatic duct, dilatated right posterior bile ducts (arrowhead), and the modest dilatation in anterior bile duct (arrow). (**F**) ^18^F-FDG PET-CT showed strong uptake at the intrahepatic tumor. (**G**) Lower uptake was observed at the lymphadenopathy in the same ^18^F-FDG PET-CT (arrow). CT, computed tomography; ERCP, endoscopic retrograde cholangiopancreatography; ^18^F-FDG PET-CT, fluorin-18 fluorodeoxyglucose positron emission tomography–computed tomography

**Fig. 2:**
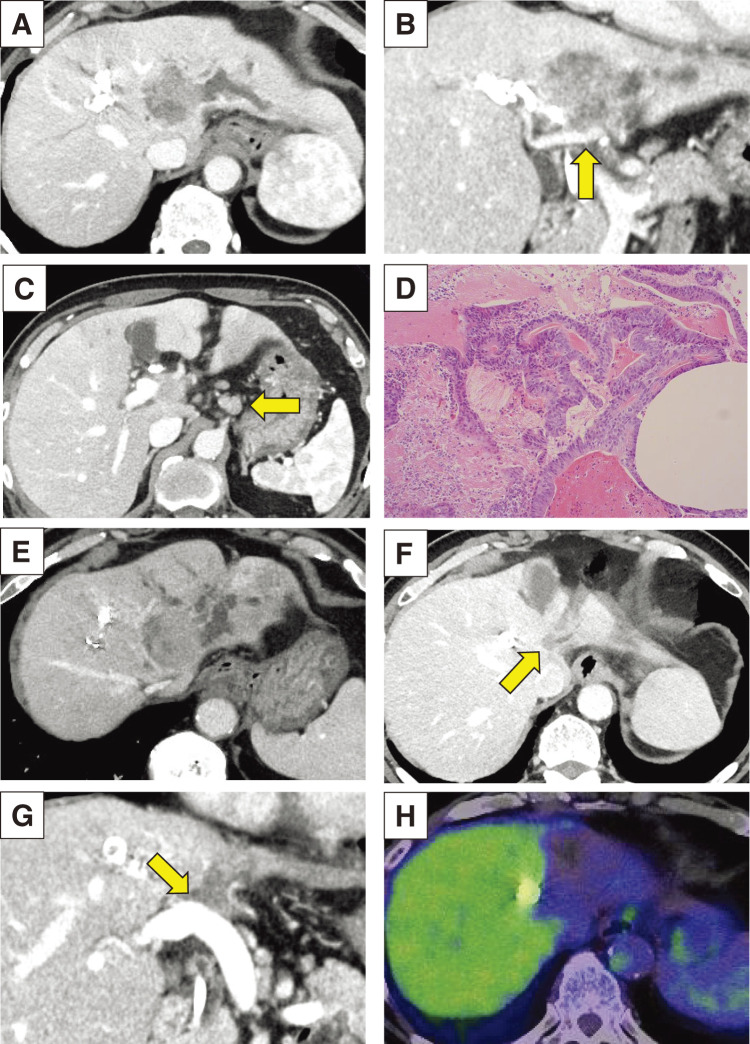
Radiologic changes of imaging findings over time. (**A**) Dynamic enhanced CT after PTPE showed tumor growth, 41 mm in diameter, during the waiting period for liver hypertrophy. (**B**) Tumor invasion to the right hepatic artery (arrow) was observed in the coronal view of the same CT. (**C**) Multiple metastasis of lymph node (arrow) was more obvious than before PTPE. (**D**) Pathological findings showed well-differentiated tubular adenocarcinoma (H&E stain, ×100). (**E**) Dynamic enhanced CT after 1st-line chemotherapy of 5 cycles of gemcitabine and cisplatin showed slight tumor growth, 42 mm in diameter. (**F**) Dynamic enhanced CT after 2nd-line treatment of pembrolizumab showed extreme tumor shrinkage, 16 mm in diameter (arrow), which kept the same size throughout the pembrolizumab therapy. (**G**) Persistent tumor invasion to the portal vein (arrow) was observed in the coronal view of the same CT after pembrolizumab therapy. (**H**) ^18^F-FDG PET-CT after pembrolizumab therapy. CT, computed tomography; PTPE, percutaneous transhepatic portal vein embolization; ^18^F-FDG PET-CT, fluorin-18 fluorodeoxyglucose positron emission tomography-CT; H&E, hematoxylin and eosin

A 69-year-old male with epigastralgia and jaundice was introduced to our hospital. Blood test showed increasing total bilirubin level of up to 2.32 mg/dL and elevation of tumor marker carbohydrate antigen 19-9 (CA19-9) up to 382 U/dL. Computed tomography (CT) imaging showed a liver mass, 27 mm in diameter, which was located around the perihilar region, invading the hilar bile duct with an upstream dilatation of the bilateral intrahepatic bile ducts (**Fig. 1A**, **1B**). The portal bifurcation as well as the left portal vein were involved, and the left liver were atrophied. The tumor was adjacent to the root of anterior branch of portal vein (**Fig. 1C**). Bulky lymphadenopathy in the hepatoduodenal ligament, around the common hepatic artery, and celiac artery were also observed (**Fig. 1D**). Endoscopic retrograde cholangiopancreatography showed discontinuation of the left hepatic duct and dilatated right anterior and posterior bile ducts (**Fig. 1E**). Posterior bile duct was connected to the left hepatic duct, and the dilatation was more significant in the posterior than in the anterior branch. Biopsy taken from the main biliary stricture confirmed histologic evidence of adenocarcinoma. Two plastic inside stents were placed in the right anterior and posterior bile ducts. Fluorin-18 fluorodeoxyglucose positron emission tomography-CT (^18^F-FDG PET-CT) showed strong uptake (maximum standardized uptake value: 15.5) at the intrahepatic tumor (**Fig. 1F**). Lower uptake (maximum standardized uptake value: 2.5) was observed in the lymphadenopathy (**Fig. 1G**). Clinical diagnosis of advanced but resectable perihilar cholangiocarcinoma (Bismuth type IV and AJCC clinical T4N1M0, stage IIIC) was made and left hepatic trisectionectomy, caudate lobectomy, bile duct resection, and portal vein resection and reconstruction (H123458-B-PV) were initially planned for definitive treatment. Percutaneous transhepatic portal vein embolization (PTPE) to the anterior portal vein was performed prior to surgery. However, the second CT to evaluate liver hypertrophy unexpectedly demonstrated a rapid tumor growth of up to 41 mm invading the right hepatic artery and multiple metastasis of lymph node (**Fig. 2A**–**2C**). Additional resection of the right hepatic artery (H123458-B-PV-HA) was also needed for curatively intended surgery. We revised our surgical decision of resectable disease to unresectable disease, considering the rapidly growing nature, progressive disease stage (cT4N2M0, stage IVA), and the extensive nature of the modified surgical procedure. Percutaneous needle biopsy from the intrahepatic tumor was performed to obtain enough amount of tumor tissue for multi-gene panel testing, histologically confirming well-differentiated tubular adenocarcinoma (**Fig. 2D**).

**Fig. 3:**
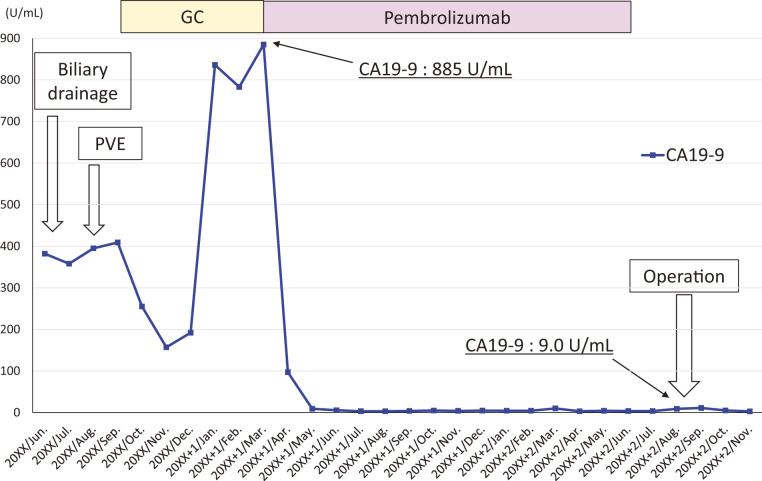
Timeline of treatment and tumor marker. GC, gemcitabine and cisplatin; PVE, portal vein embolization; CA19-9, carbohydrate antigen 19-9

**Fig. 4:**
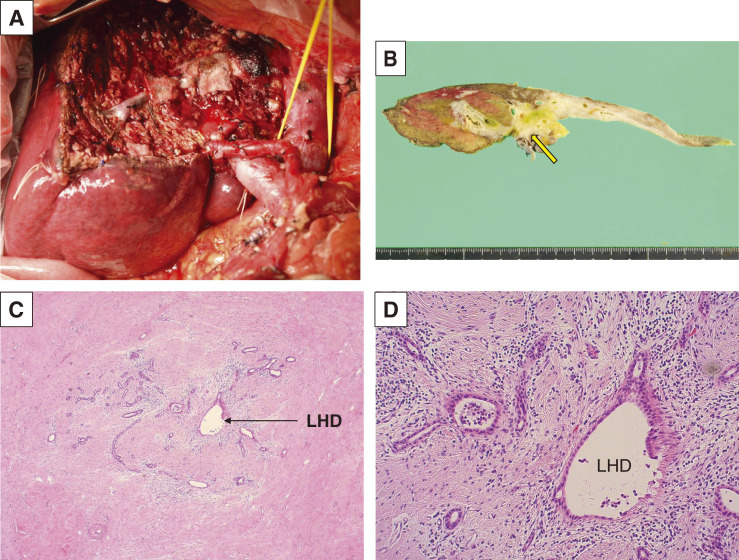
Findings of conversion surgery. (**A**) Operative findings after resection; left trisectionectomy and caudate lobectomy with portal vein resection and reconstruction. (**B**) Gross finding of the resected specimen showed white area indicating previous existence of tumor (arrow). (**C**, **D**) The pathological findings around left hepatic duct. There were no residual tumor cells, whereas invasion of inflammatory cells and fibrosis were observed. (**C**): ×25, (**D**): ×100. LHD, left hepatic duct

The patient underwent systemic chemotherapy of gemcitabine and cisplatin as first-line, and showed slight tumor progression ranging the status of stable disease after 5 cycles of administration (**Fig. 2E**) with re-elevation of CA19-9 (pre-chemotherapy: 409 U/dL, after 2 cycles: 157 U/dL, after 5 cycles: 885 U/dL). Because the cancer multi-gene panel testing using percutaneous tissue sample revealed MSI-high and TMB-high, whereas no fibroblast growth factor receptor 2 (FGFR2)-related abnormality, he received pembrolizumab monotherapy as second-line therapy. The tumor size reduced drastically by over 30% and the tumor marker CA19-9 decreased within the normal level only after 2 cycles of administration, providing a clinical partial response. After continuous tumor control during 22 cycles of pembrolizumab (approximately 15 month-period) with only minor adverse events (dermatitis; grade 1 and interstitial pneumonia; grade 1), the tumor became extremely small, 16 mm in diameter, though it did not disappear (**Fig. 2F**) showing persistent invasion to the portal vein (**Fig. 2G**). PET-CT was performed again, showing no uptake in the tumor (**Fig. 2H**). The CA19-9 during this period kept normal (**Fig. 3**). Here, we planned left trisectionectomy and caudate lobectomy with portal vein resection/reconstruction and possible resection of the right hepatic artery as conversion surgery, about 26 months from the initial presentation. On laparotomy, the left and anterior lobe were extensively shrunk and a tumor scar was observed in the left hilar plate; however, there was no hard tumor palpable. Several enlarged lymph nodes including para-aortic nodes were sampled for intraoperative frozen section diagnosis, showing no evidence of metastasis. In addition, the right hepatic artery was extensively involved with fibrosis, but fully isolated from the bile duct with no evidence of malignancy of the surrounding fibrotic tissue. Thus, resection was completed without hepatic artery resection (**Fig. 4A**). Operation time was 748 minutes and blood loss was up to 1118 mL. Gross inspection of the resected specimen showed that the whitish fibrotic area indicated the previous tumor mass (**Fig. 4B**). Pathologically (**Fig. 4C**, **4D**), there were no residual cancer cells neither in the primary site nor in the regional lymph nodes, giving pathological complete response (ypT0N0M0). The patient suffered from bile leakage and was discharged on postoperative day 31. He was followed up without adjuvant chemotherapy, and was alive without disease 4 months after resection.

## DISCUSSION

The results of the KEYNOTE-158 trial have promoted that pembrolizumab therapy for MSI-high or TMB-high solid tumors is a standard treatment across various tumor origins.^9,10)^ Among the 233 study patients with MSI-high unresectable cancer, those with BTC accounted for only 9.4% (*n* = 22) and they radiologically demonstrated a good objective response rate of 40.9%, including 2 patients with complete response after pembrolizumab monotherapy.^9)^ However, there is no available data about pCR in this trial.^9)^ Real-world data reported that the MSI-high status was found in only 2%–9% among the whole BTC^11,12)^; conversely, BTC accounted for only 2.4% of the total MSI-high cancers.^11)^ This small proportion can be explained by 2 major factors. First, BTC is a globally rare disease; second, there is a BTC-specific problem that satisfactory cancer sample volume for assessment of MSI status is difficult or sometimes impossible to gain in the unresectable setting.^13)^ In addition, histologic efficacy of chemotherapy should be evaluated not by biopsy tissue sample but by the whole tumor sampling to avoid overestimation of CR. Therefore, pCR associated with pembrolizumab treatment against MSI-high BTC is difficult to prove in practice.

Some high-volume centers reported the experience of conversion surgery after systemic chemotherapy for initially unresectable BTC, and there seemed to be pCR patients although very limited.^14–17)^ However, the chemotherapy in these patients used cytotoxic agents alone represented by gemcitabine/cisplatin, gemcitabine/S-1, or gemcitabine/cisplatin/S-1. Wang et al. compared conversion surgery and continued systemic therapy including PD-1/PD-L1 (programmed cell death ligand 1) inhibitor for unresectable BTC,^18)^ in which 13 patients underwent conversion surgery and 2 patients (15.4%) achieved pCR. Although this study included patients with pembrolizumab, there is no statement about MSI status. In addition, there have been several case reports that proved pCR after conversion surgery in BTC.^19–25)^ These literatures in the past decade suggested that a small proportion of patients with initially unresectable BTC may have a chance of conversion surgery, and a minute subset of them might have no viable tumor cells. Ideally, the status of pCR patients should be radiologically diagnosed to avoid unnecessary resection in the future.

Promising outcomes of pembrolizumab treatment are reported in MSI- or TMB-high BTC,^13,26–30)^ however, pCR status in the final evaluation are very limited. Abudalou et al. reported a case of pCR after 5 cycles of pembrolizumab monotherapy for TMB-high intrahepatic cholangiocarcinoma removed by extended left hepatectomy with inferior vena cava and right hepatic vein reconstruction.^31)^ Robinson et al. reported a TMB-high case of intrahepatic cholangiocarcinoma who underwent 2 cycles of pembrolizumab monotherapy followed by resection with right anterior sectionectomy with right adrenalectomy, whose pathological finding was pCR.^32)^ Compared with recent triplet chemotherapy for BTC including gemcitabine, cisplatin, and durvalumab (GCD)^5)^ and gemcitabine, cisplatin, and pembrolizumab (GCP)^7)^), pembrolizumab monotherapy limited for MSI-high BTC showed a relatively better overall response rate (40.9%) and overall survival time (24.3 months)^9)^ (GCD: 26.7% and 12.8 months,^5)^ GCP: 28.7%, and 12.7 months^7)^). Thus, pembrolizumab for MSI- or TMB-high BTC may have a high potential of pCR even in far advanced BTC.

Recently, non-operative management with PD-1 blockade (watch-and-wait strategy) has been discussed in MSI-high locally advanced rectal cancer.^33–37)^ This approach can be realized because of high clinical complete response (cCR) rates and high pCR rate in the limited subpopulation of rectal cancer.^34,35)^ In addition, definitive resection for advanced tumor carries frequent complications and impairs quality of life, driving the debate about avoidance of surgery.^34)^ BTC, particularly perihilar cholangiocarcinoma, often requires complicated hepatectomy with a high mortality rate in 5% in Japan and >15% in the West. The wait-and-see policy using pembrolizumab monotherapy may be a promising option, provided that clinical partial response is observed in MSI-high BTC.

## CONCLUSIONS

We experienced pCR after pembrolizumab as second-line therapy for MSI-high perihilar cholangiocarcinoma. Conversion surgery should be considered in select patients who demonstrate a significant downsize or downstage by pembrolizumab. Non-surgical management, however, may be possible in BTC to avoid extended surgical resection.

## DECLARATIONS

### Funding

This work was not supported by any fundings.

### Authors' contributions

YI and TE conceived and designed the case reports and wrote the main manuscript.

YI, HM, MY, SO, and TE obtained informed consent from the patient and performed the surgery.

SK, NW, and TM participated in perioperative patient management.

KO and FO conducted systemic drug therapies.

YI, MY, SK, NW, SO, TM, KO, FO, MK, and TE critically reviewed the manuscript.

All the authors have read and approved the final manuscript.

### Availability of data and materials

The datasets of this case report are available from the corresponding author upon reasonable request.

### Ethics approval and consent to participate

Not applicable.

### Consent for publication

Informed consent was obtained from the patient for the publication of this case report and accompanying images.

### Competing interests

TE has received personal fees from Taiho Pharmaceutical and AstraZeneca. The other authors declare that they have no competing interests.
